# A Rare Presentation of Colon Carcinoma Metastasis Within a Meningioma: A Case Report and Literature Review

**DOI:** 10.7759/cureus.45764

**Published:** 2023-09-22

**Authors:** Salman J Khan, FNU Anum, FNU Vishal, Michael Lee, Seemab Sheikh, Syed Asjad Tauheed Zaidi, Syeda Fatima Murtaza, Vinod Kumar

**Affiliations:** 1 Public Health, University of Massachusetts Amherst, Amherst, USA; 2 Hematology and Oncology, Mayo Clinic, Jacksonville, USA; 3 Internal Medicine, Peoples University of Medical & Health Science for Women, Nawabshah, PAK; 4 Infectious Diseases, Johns Hopkins Bloomberg School of Public Health, Baltimore, USA; 5 Infectious Diseases, Rochester Regional Health, Rochester, USA; 6 Internal Medicine, Jefferson Health, Philadelphia, USA; 7 Internal Medicine, Community Health Center, New Britain, USA; 8 Internal Medicine, Monmouth Medical Center, Long Branch, USA; 9 Medicine, Shalamar Medical and Dental College, Lahore, PAK; 10 Medicine, Mayo Clinic, Jacksonville, USA; 11 Medicine, Allama Iqbal Medical College, Lahore, PAK

**Keywords:** metastasis, colon adenocarcinoma within meningioma, meningioma, colon cancer, tumor-to-tumor metastasis

## Abstract

Colon carcinoma with brain metastasis is a rare presentation. This presentation is more unusual and unique when the single brain metastatic lesion has two different types of tumors. This rare phenomenon is known as a tumor-to-tumor metastasis. A meningioma usually hosts lung and breast cancers within it. However, colon carcinoma metastasis into meningiomas has rarely been reported. An 86-year-old man presented with neurological symptoms and was found to have a brain mass. The search for primary lesions was negative as the chest, abdomen, and pelvis CT scan was insignificant. When the brain lesion’s pathology revealed a composite mass of adenocarcinoma and a meningioma, further investigation with a colonoscopy revealed a colonic mass as the primary metastasis lesion. This unique presentation and pathology emphasize the importance of a comprehensive investigative approach to finding the primary lesions and consideration of such a phenomenon in these lesions.

## Introduction

A rare phenomenon of tumor-to-tumor metastasis occurs in some patients in which one cancer increases the risk of metastasis of other cancer within it. Patients with meningiomas have exhibited this phenomenon. Mainly, lung or breast cancer patients have been reported to have metastasis within meningiomas. However, colon carcinoma metastasizing within a meningioma is extremely rare. Only one case has been reported so far. In such cases, diagnosing such a dual-cancer presence is the most critical factor affecting the management course. In this article, we present the case of a patient with a right temporal mass, which was later found to be metastatic colon cancer and was growing within a meningioma, along with the literature review of such a rare case.

## Case presentation

An 86-year-old man presented to the emergency department with left upper extremity (LUE) weakness, left facial droop, and headaches. He had been experiencing generalized weakness for the past six months, but for the past two days, symptoms worsened with focused weakness in the LUE.

On physical examination, he had impaired sensations in the LUE. The strength was 5/5 on the right and 2/5 on the left side. A computed tomography (CT) scan of the head was done, which showed a large heterogeneous mass in the right temporal region measuring 6.2 cm. The patient was admitted to the ICU. 

**Figure 1:**
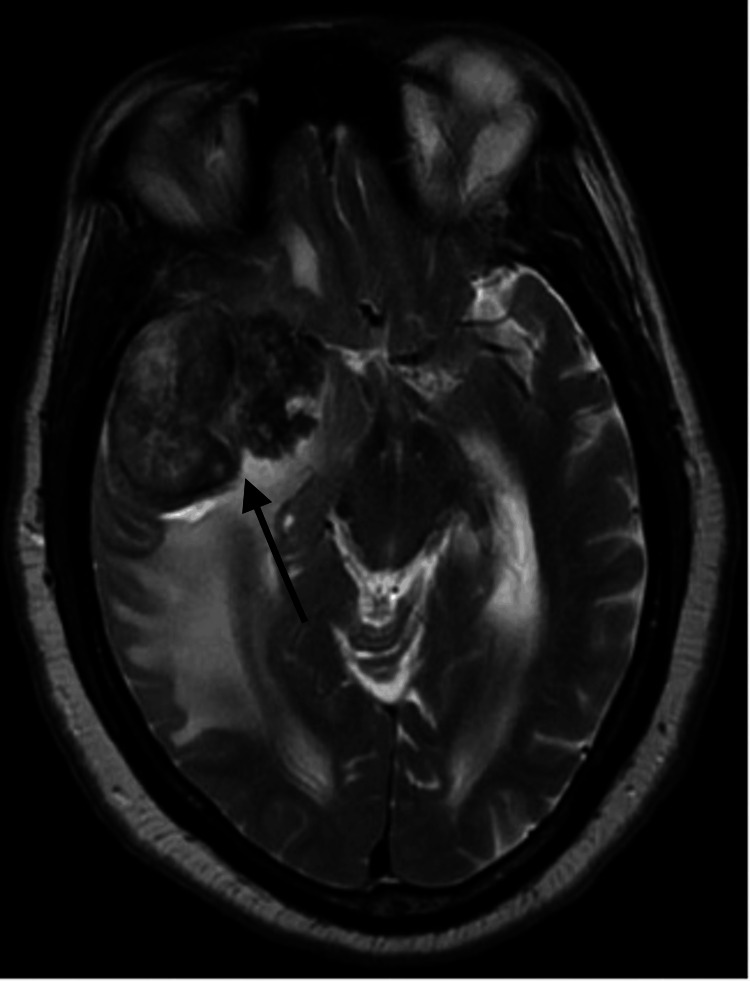
4.1 x 5.3 x 5.8 cm right frontotemporal mass with a 1.2 cm left shift.

**Figure 2:**
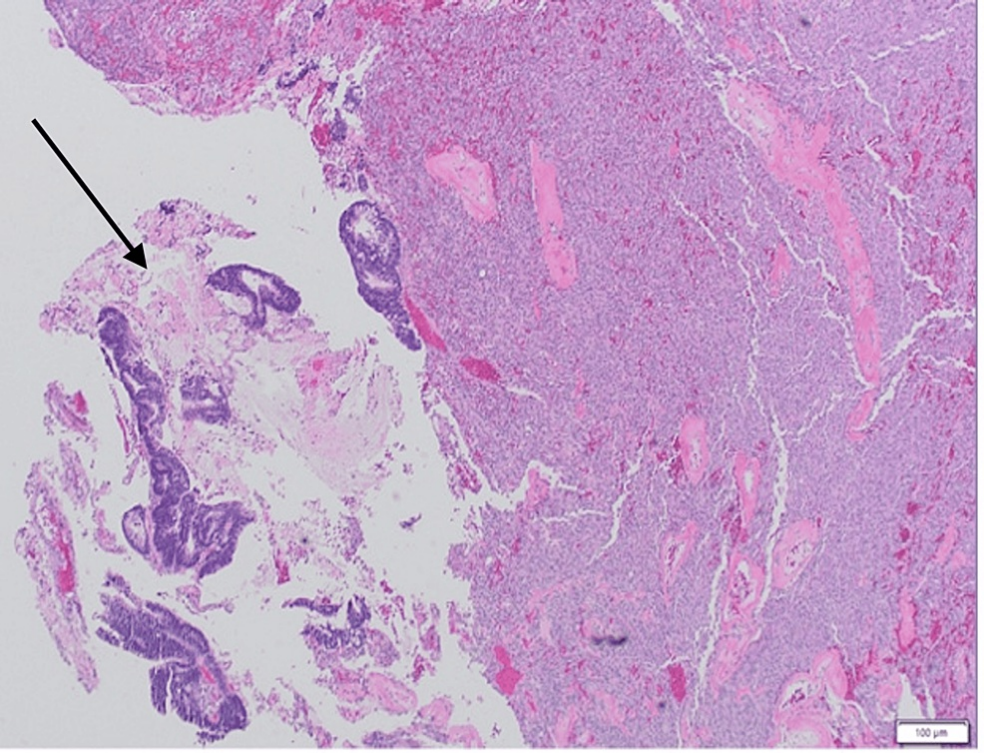
Moderately differentiated adenocarcinoma consisting of branching and fused glands with a visible lumen on the left side of the figure. The adenocarcinoma consists of cells with hyperchromatic nuclei and irregular nuclear borders. The meningioma is shown on the mid to right side of the figure, which is hypercellular and contains medium-sized cells with eosinophilic cytoplasm, round to oval nuclei with inconspicuous nucleoli, and indistinct cell borders.

CT chest, abdomen, and pelvis (CAP) were done to confirm any primary lesion for further management. It was negative for any other source of brain mass. At this point, we were thinking of primary brain lesions, as there were no lesions seen on the CT CAP that could be the cause of the brain mass. MRI was done, and it showed a 4.1 x 5.3 x 5.8 cm right frontotemporal mass with a 1.2 cm left shift. The mass did show some peripheral hemorrhagic components (Figure 1). Right pterional craniotomy was done three days later for the tumor resection of the temporal lobe. We believed that it was the primary brain lesion; hence, the diagnosis of glioblastoma was considered. However, the pathology report changed the perspective, which revealed predominantly metastatic adenocarcinoma with a meningioma (Figure 2).

Immunohistochemical staining revealed that the carcinoma was positive for CK20 and CDX2 and negative for CK7, TTF-1, GATA3, and PSA. Staining showed that the meningioma is positive for epithelial membrane antigen (EMA) and progesterone receptor (PR). Ki-67 showed a proliferation index of approximately 2%. 

**Figure 3:**
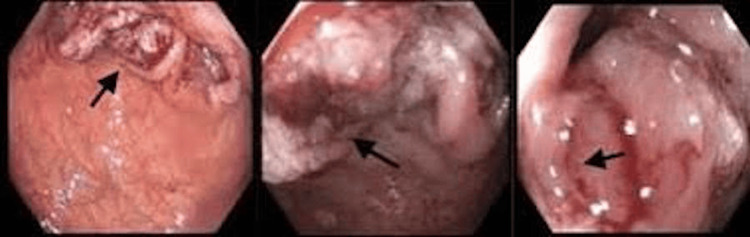
Colonoscopy images showing a large mass at the splenic flexure.

The patient was then considered for further investigation to determine the metastatic brain lesion. A gastrointestinal consult was taken. He had a colonoscopy done more than 10 years ago. There was no history of colon cancer. However, upon extensive multidisciplinary discussions, he underwent a colonoscopy, and a large mass measuring 6 × 4 × 2.6 cm at splenic flexure was found (Figure 3).

General surgery was consulted, and he underwent robotic resection of the splenic flexure with the da Vinci® XI Robotic Surgical System (Intuitive Surgical Inc., USA). The colonic mass pathology showed grade 2 adenocarcinoma invading the visceral peritoneum and regional lymph nodes. Genetic testing was done, and it came back negative. Although the patient needed adjuvant chemotherapy because of an aggressive and metastatic disease, he refused further management. The patient was then referred to hospice.

## Discussion

A tumor-to-tumor metastasis is a rare phenomenon that involves the occurrence of two cancers within the same mass. One is the primary cancer that hosts, and the other is metastatic cancer [1]. The most common neoplasms seen as the host are renal cell carcinomas, meningiomas, thyroid cancers, pituitary adenomas, and gliomas. The most common donor metastatic cancers in this phenomenon are lung, breast, prostate, and malignant melanomas [2]. A true tumor-to-tumor metastasis must meet specific criteria that include the existence of more than one primary tumor; the host tumor must be a true benign or malignant neoplasm; metastatic cancer must be a true metastatic neoplasm employing the true mode of metastasis, not just a contiguous growth; and the neoplasm must not metastasize to the lymphatic system [3].

Many cases have been reported where the meningioma acted as the host neoplasm. Several factors have been studied to be the reason for this favorability for meningiomas by donor metastatic cancers. Rich vascular network, low metabolic rate, high collagen and lipid content, and E-cadherin immunoreactivity in meningiomas make them favorable and noncompetitive destinations for metastatic cancers [4]. However, most cases have breast and lung cancers as donor neoplasms. 

Upon searching the literature on PubMed, Google Scholar, and Web of Science, only one case of colon carcinoma as donor cancer metastasizing to a meningioma has been reported so far (Table 1) [5]. In this case, a metastatic lesion was suspected due to a previous history of colon carcinoma. Nonetheless, tumor-to-tumor metastasis was never considered because of a lack of evidence for colon carcinoma being a donor in the past. By contrast, our patient had no cancer or family history. Moreover, he was consistent with colon carcinoma screening colonoscopies till the age of 75, which were negative. These facts make our case a unique addition to the literature as an example of considering colon carcinoma as a tumor-to-tumor metastasis under such circumstances. 

**Table 1:** A case of tumor-to-tumor metastasis involving a meningioma and colon carcinoma based on the literature review Reference: [5]

	Patient’s age	Cancer history	Meningioma with colon carcinoma metastasis	Histology
	76 years	Yes	Confirmed by biopsy	Uniform elongated cells occasionally forming lobules showing oval nuclei with frequent clear inclusions

Being a rare presentation, the management of these cases is challenging. The investigation to look for primary lesions in patients with a brain mass has immense importance and must always be thorough. However, radiological findings cannot be specific or sensitive for tumor-to-tumor metastasis as CT scans or MRI cannot reliably exclude the metastasis within meningiomas. Another technique that can increase the chances of considering metastasis within meningiomas is proton spectroscopic MRI (sMRI) [6]. It evaluates the aggressiveness of meningiomas by looking for the metabolic composition of the mass. The suspicion can be raised with the lactate/creatinine ratio increase. Although sMRI can help diagnose meningiomas with metastasis, it cannot surpass the histopathological analysis of the mass lesion. There is a need for research regarding more preoperative diagnostic techniques to diagnose tumor-to-tumor metastasis involving meningiomas. Furthermore, even when the CT scan is negative, colon carcinoma should not be ruled out unless a colonoscopy reveals no lesions. Taking these points into consideration can change the management of the patient, which can improve the patient's outcome.

## Conclusions

Tumor-to-tumor metastasis involving meningiomas is a rare phenomenon. Mostly, lung and breast cancers have been reported as donor metastasis in it. Colon carcinoma metastasis within meningiomas is a unique and rare presentation. Our patient presented with a brain lesion found to be colon carcinoma metastasis within a meningioma. He had no history of cancer. Traditional investigation tests, such as CT, showed no colon lesion. A more thorough approach was chosen to go for a colonoscopy, and a colonic mass was finally found. There is a need for more definitive investigations for metastasis within meningiomas. Colon carcinoma should also be considered in patients with tumor-to-tumor metastasis.
